# Seafood Consumption and Its Contribution to Nutrients Intake among Canadians in 2004 and 2015

**DOI:** 10.3390/nu13010077

**Published:** 2020-12-29

**Authors:** Xue Feng Hu, Hing Man Chan

**Affiliations:** Department of Biology, University of Ottawa (XFH, HMC), Ottawa, ON K1N 6N5, Canada; xuefeng.hu@uottawa.ca

**Keywords:** seafood, fish, shellfish, nutrients, Canada, Canadian Community Health Survey

## Abstract

Fish and seafood are excellent sources of nutrients such as omega-3 fatty acids, Vitamin D, and selenium. The aims of this study were to examine the pattern of seafood consumption among Canadians and determine their contribution to intakes of energy and nutrients. Day-1 24-h dietary recalls data collected from a national survey, the Canadian Community Health Survey—Nutrition in 2004 and 2015, were used to estimate food and nutrient intakes among Canadians. Seafood was classified according to the Bureau of Nutritional Sciences food list. Descriptive statistics were used to calculate the consumption rate and the average consumption amount of seafood by different age groups and sociodemographic characteristics. Population ratios were used to assess the contribution of seafood to the total intake of energy and nutrients. The overall consumption rate of seafood was around 17%, and the rate was similar between males and females, and slightly higher in 2015 (17.71%) compared to 2004 (16.38%). The average portion size is approximately 100 g, which translates into a ≈36 kg annual intake among the consumers and ≈6.2 kg per capita consumption. Adults (especially 30 years and above), Asians, individuals who were married, and with post-secondary education were more likely to consume seafood. Salmon, tuna, shrimp, cod, and crab were the most frequently consumed seafood in Canada, the consumption rate of which all increased from 2004 to 2015. Seafood provided up to 75% of *n*-3 PUFAs, 18% of Vitamin D, 19% Vitamin B12, 6% of niacin, and 4% of Vitamin B6 from all food sources. Seafood consumers had a healthier diet, as seafood consumption was related to a higher intake of key nutrients and a lower intake of total sugar and saturated fatty acids. Therefore, fish consumption should be promoted among Canadians.

## 1. Introduction

Fish and seafood are an excellent source of docosahexaenoic acid (DHA), eicosapentaenoic acid (EPA), Vitamin D, selenium, and other nutrients [1,2,3]. Emerging evidence from observational and experimental studies and randomized controlled trials suggests that fish consumption and the associated intake of *n*-3 PUFAs can help maintain cardiovascular health [1,2,3]. Fish consumption and fish oil supplementation have been shown to be related to lower blood pressure, plasma triglycerides, resting heart rate, and inflammation and the improvement of myocardial filling and efficiency and vascular function [1,4]. Evidence also suggests that regular fish consumption or fish oil supplementation during pregnancy may have potentially beneficial effects on the child’s cognitive development [5,6].

The 2015–2020 Dietary Guidelines for Americans recommends at least eight ounces of fish and seafood (less for young children) per week based on a 2000-calorie diet [7]. Canada’s Food Guide 2017 recommends choosing plenty of protein foods and foods with healthy fats instead of saturated fat [8]. Seafood is a good example of protein foods as part of a healthy diet. The previous 2007 Canada’s Food Guide recommends at least two servings (75 g each) of fish a week [9]. However, limited information is available on recent national fish and seafood intake in Canada. National food expenditure and food supply data on fish and seafood are available in Canada. The average annual household spending on fish and seafood was 219 Canadian dollars in 2017, which accounted for 2.6% of total food expenditure [10]. Fisheries and Oceans Canada estimated that the fish products available are 9.14 kg per capita, and the fish consumption will increase by 11% by 2025 [11]. The estimated average intake of finfish in the early 1990s was 22 g/day, i.e., 8.03 kg per year among adult consumers in Canada [12]. These numbers need to be verified and updated with fish consumption data independently.

There is no literature available describing the temporal trend for fish consumption among Canadians over the years, in light of recent evidence of beneficial health effects of fish and *n*-3 PUFAs. The objectives of the present study were to (1) examine changes in seafood consumption among Canadians over time with data collected from nationally representative nutrition surveys conducted in 2004 and 2015, (2) investigate the sociodemographic factors associated with seafood consumption, (3) examine the differences in energy and nutrients intake between consumers and non-consumers of seafood, and (4) assess the contribution of seafood to total nutrients intake among the consumers.

## 2. Materials and Methods

### CCHS 2004 and 2015

The Canadian Community Health Survey (CCHS) is a series of nationally representative cross-sectional surveys with a different focus in each of its cycles. The common components of each cycle collect information on health status, health care utilization, and health determinants for the Canadian population [13]. The CCHS 2004-Nutrition (previously called CCHS cycle 2.2) and CCHS 2015-Nutrition are the two cycles that focus on nutrition. Both surveys were composed of two parts: a general health questionnaire and a 24-h dietary recall. The general health component collects information on respondents’ sociodemographic characteristics (e.g., age, sex, marital status, education, and income), chronic health conditions, and use of vitamin and mineral supplements. The 24-h recall gathers information on foods and beverages consumed via a computer-assisted personal interview for the total sample (day 1) and a telephonic interview on sub-sample (day 2). Both surveys covered Canadians living within the ten provinces. Individuals living on reserves or Indigenous settlements, full-time members of the Canadian forces, and the institutionalized population were excluded from the survey.

This study included respondents who were two years of age and older. Pregnant and lactating women, children who consumed milk purely, individuals who did not report any food item in the 24-h recall, and individuals with <400 kcal and >4000 kcal energy intake were excluded. Data from the Day 1 recalls were used, as only about 1/3 of the respondents completed a Day 2 recall. The final sample sizes of Day 1 recall from both surveys (*n* = 27,816 in 2002 and *n* = 19,273 in 2015) represent approximately 98% of the Canadian population. Nutrient intakes from food sources only were used to estimate the contribution of nutrients and energy from seafood, i.e., supplements were excluded.

## 3. Data File Structure and Food Classification

Sociodemographic information and total nutrient intakes from food sources from Day 1 recall were provided in the health component, vitamin, and mineral supplements, 24-h dietary recall (HS) file. Nutrient values for all items reported in the 24-h dietary recalls were provided in the food and ingredient details (FID) file. Nutrients intake from each seafood species was the product of intake amount and nutrients value of that seafood species. Total nutrients intake from seafood was the sum of nutrients from all seafood species for each individual. Fish (group code 34) and shellfish (group code 35) were defined using the food group list from the Bureau of Nutritional Sciences (BNS) [14]. In the manuscript hence after, the term “seafood” refers to finfish and shellfish combined. Seafood species were identified through food codes and food names from the Canadian Nutrient File [15]. For the top 10 consumed seafood species, the total intake amount for all sub-species (e.g., salmon, Atlantic/chinook), wild or farmed, and preparation methods (raw or cooked) were reported.

## 4. Statistical Analysis

The prevalence of seafood consumption (used interchangeably with seafood consumption rate) was summarized by the following sociodemographic characteristics: marital status (married or not), ethnic groups (Caucasians, Asians, Other), education (with or without post-secondary education), dwelling regions (Coastal regions or not), and household income (below or above $60,000 CAD/year, i.e., approximately the median income). Coastal regions were defined as British Columbia, New Brunswick, Newfoundland and Labrador, Nova Scotia, Prince Edward Island, and Quebec. The prevalence of seafood consumers and average consumption among consumers were summarized by age groups (two to three years, four to eight years, nine to 13 years, 14 to 18 years, 19 to 30 years, 31 to 50 years, 51 to 70 years, 71 years and above) and by seafood species. Energy and nutrients intake were summarized by seafood consumers and non-consumers. The population ratio method was adopted to calculate the percentage contribution of nutrients and energy from seafood. The intake of a given nutrient (e.g., Vitamin D) provided by seafood was summed across all individuals and divided by the total intake of that nutrient for all individuals. This method has been shown to provide better estimates of population usual intakes in contrast to other methods, e.g., averaging the ratio calculated at the individual level [16]. The population ratio was calculated between the total nutrients and energy intake from seafood from all consumers (FID file) and the total nutrients and energy intake from all respondents (HS file).

All analyses were performed using Stata SE^®^ (version 16, StataCorp, College Station, TX, USA). Sample weights and 500 bootstrap weights were used to adjust for sampling design, generate population-representative statistics, and produce appropriate variance estimation. Data were shown as mean ± standard error. All analyses were performed separately for males and females. The term “higher/more” or “lower/less” in the Section 5 refers to a statistically significant difference in percentage or mean unless specified otherwise. Significance was defined at *p* < 0.05 after Bonferroni correction for multiple comparisons.

## 5. Results

Consumption of seafood according to different sociodemographic characteristics is presented in Table 1. On a given day, 16.38% of the Canadians reported consuming seafood in 2004 and 17.71% in 2015, respectively. No difference was observed in the prevalence of seafood consumption between males and females in 2004 and 2015. Married individuals were more likely to consume seafood compared to other marital statuses (18.61% vs. 14.22% in 2004, and 18.83% vs. 16.52% in 2015, *p* < 0.05). Asians and individuals with other ethnic backgrounds were more likely to consume seafood compared to Caucasians. The prevalence of seafood consumption was similar for Caucasians from 2004 (14.62%) to 2015 (15.43%), while it decreased among Asians (from 31.97% to 24.05%, *p* < 0.05) but increased among individuals with other ethnic backgrounds (from 17.62% to 22.89%, *p* < 0.05). Individuals with post-secondary education were more likely to consume seafood compared to individuals without (19.26% vs. 13.55% in 2004, and 20.05% vs. 15.95% in 2015, *p* < 0.05). The prevalence of seafood consumption was slightly higher among individuals dwelling in coastal provinces (17.42% vs. 15.51% in 2004 and 19.16% vs. 16.61% in 2015, *p* < 0.05). No significant difference was observed for individuals with annual household income below and over $60,000.

Seafood consumption among age groups is presented in Table 2. Among the consumers on a given day, the average daily intake was approximately 100 g (108.02 g in 2004 and 97.69 g in 2015). The corresponding daily consumption per capita was 17.66 g in 2004 and 17.33 g in 2015. Adults, especially individuals aged 31 years and above, were more likely to consume seafood compared to children in both 2004 and 2015. The prevalence of seafood consumption was stable in adult females and most age groups in males in 2004 and 2015. The most significant increase in the prevalence of seafood consumption was observed among children (two to three years old) from 2004 to 2015 (12.74% to 21.74% in males and 13.85% to 17.66% in females, *p* < 0.05). The prevalence of seafood consumption increased in female children of all age groups from 2004 to 2015. Males consumed more seafood than females (on average 20 to 40 g) in both 2004 and 2015. The average daily intake decreased among individuals aged 31 years and above (on average 20 g), while it increased among individuals aged 14 to 30 years old (on average 20 g).

Seafood consumption by species is shown in Table 3. Salmon, tuna, shrimp, cod, and crab were the top five consumed seafood in both 2004 and 2015. The prevalence of consumption increased by more than 1% for salmon and cod and by nearly 1% for shrimp and crab from 2004 to 2015. The consumption rate for tuna, sardine, trout, herring, clam, and scallop was relatively stable in 2004 and 2015. Among the consumers on a given day, the average daily consumption decreased by 20 to 40 g for most of the species except for sardine and herring from 2004 to 2015. Males tended to consume more for most species in 2004, while the daily consumption was more equal between males and females in 2015.

Daily energy and nutrients intake between consumers and non-consumers of seafood on a given day are presented in Table 4. Respondents to CCHS 2015 reported lower energy and nutrients intake compared to CCHS 2004. Consumers and non-consumers of seafood on a given day had similar total energy intake between males and females in both 2004 and 2015. On a given day, seafood consumers showed a healthier diet pattern compared to the non-consumers in both years. For example, seafood consumers had a lower intake of total sugar (≈8 g/day) and saturated fat (≈2 g/day), and a higher intake of multiple key nutrients, e.g., protein (≈10 g/day), polyunsaturated fatty acid (≈2 g/day), Vitamin A (≈60 µg/day), Vitamin D (≈5 µg/day), Vitamin B12 (≈3 µg/day), natural occurring folate (≈30 µg/day), magnesium (≈40 µg/day), etc. The differences in nutrients intake were smaller in 2015 than in 2004.

The percentage contribution of seafood to total energy and nutrients intake is presented in Table 5. The percentage contribution from seafood to total energy and nutrients intake was similar between male and female and between 2004 and 2015. Seafood provided more energy and nutrients intake in adults compared to children. Overall, seafood consumption contributed ≈ 0.5% (varied from 0.3% to 0.7%) of the total weight of food consumed, and ≈1% (varied from 0.5% to 1.5%) of the total energy intake. However, seafood provided up to ≈ 6% of protein, ≈18% of Vitamin D, ≈19% Vitamin B12, ≈6% of niacin, and ≈4% of Vitamin B6 from food sources. Seafood was also a major contributor to omega-3 fatty acids from food sources.

## 6. Discussion

The present study reports dietary seafood intake and its contribution to nutrients intake in the Canadian population in 2004 and 2015 through nationally representative survey data. The overall consumption rate of seafood was around 17%, or one in six Canadians reported eating seafood on a given day. The consumption rate was similar to the nutrition survey conducted in Quebec in 1992 but lower than a similar survey conducted in Nova Scotia in 1993 [12]. A similar consumption rate was reported in the United States and Australia [17,18]. The average daily consumption is approximately 100 g, which translates into a ≈36 kg annual intake among the consumers and ≈6.2 kg per capita consumption.

The per capita consumption is lower compared to the Canada Food Statistics 2002 (7.2 kg) [12] but is very close to the seafood consumption data in Quebec and Nova Scotia in the 1990s [12] and in the United States [19,20]. The rate was lower than in some countries where people are habituated to seafood, e.g., China and Japan [21,22]. This may explain why the seafood consumption rate was much higher among respondents with Asian ethnic backgrounds. This ethnic-specific factor was also reported in the United States, where the consumption rate among Non-Hispanic Asians (41.2%) was twice as that of Non-Hispanic whites (18.7%) [20]. A noticeable decrease in seafood consumption rate was observed for Asians from 2004 to 2015, although the reason was not apparent. Individuals with higher education tended to eat more seafood. One plausible explanation was that Asian respondents had a higher consumption rate and relatively higher education levels concurrently. It is surprising that the seafood consumption rate was very similar among coastal provinces and inland provinces. That may reflect the fact that a large proportion of the seafood consumed among Canadians was either frozen or processed [23].

The previous version of Canada’s Food Guide used to recommend Canadians to consume at least 150 g of cooked fish each week as part of a healthy pattern of eating [9]. Our results showed that the average Canadians did not meet this consumption level (6.2 kg vs. 7.8 kg per year). The current version of Canada’s Food Guide issued in 2017 no longer has this recommendation [8]. Therefore, the consumption of fish may be further promoted by health professionals.

It is important to understand the relationships between demographics and socio-economics factors and fish consumption to target promotion efforts. The present study revealed two interesting changes in seafood consumption patterns among Canadians. First, the consumption rate increased in children from 2004 to 2015, particularly for those aged two to three years old, and for girls of all age groups. Whilst the consumption rate remained stable among adults. Second, with more respondents reported consuming seafood in 2015, the average portion size decreased by about one quarter serving (20 g), mostly from individuals aged 31 years and above. The two findings together suggested that seafood had become more popular among Canadians, and the distribution of seafood consumption had become more balanced among different population subgroups. Vegetarian diets or plant-based diets are becoming more and more popular in Canada, especially among youth and young adults [23]. Fish and seafood is an option for protein in some types of semi- and flexible vegetarian diets. That may partially explain why fish and seafood are gaining popularity among Canadian youth.

One key finding of this study is that seafood consumers showed a better nutrients intake profile than non-consumers. Seafood consumers had a lower intake of carbohydrates, total sugar, and saturated fat, and at the same time, a higher intake of multiple key nutrients, e.g., PUFAs, Vitamin A, Vitamin D, Vitamin B12, and other nutrients like phosphorus and magnesium. On the one hand, seafood is an excellent source of such nutrients. It provides up to 20% of such nutrients in a certain age group, with less than 1% of total food weight. On the other hand, fish and seafood consumption may be associated with an overall healthier dietary pattern, which contributes to better nutrients intake as well.

Due to the sampling framework of the CCHS and the present study’s analytical scope, several population groups were not covered. The current analysis provided a comprehensive picture of the seafood consumption of Canadians living in the ten provinces. First Nations living on reserve and Inuit living on the Inuit Nunangat were not included. They typically consume more fish as part of their traditional diet [24,25]. In general, the nutritional and cultural importance of fish and seafood to them and the concern of potential health impact from contaminants in fish and seafood were more crucial. Fish advisories have different recommendations for pregnant and lactating women from the general population. This group was excluded from the analysis, as the sample size was not enough to yield reliable estimates, especially subgroup analysis and fish species analysis.

There are several strengths of the present study. Both CCHS 2004 and CCHS 2015 are nationally representative surveys with comprehensive information on seafood consumption. Our results provided consumption rate and consumption per capita data for the Canadians for the first time, which could serve as baseline information for future research and policy consideration. Second, we provided detailed consumption data on seafood species. Third, CCHS has rich information on participants’ sociodemographic characteristics that allow us to reveal the change in seafood consumption patterns from 2004 to 2015 and estimated the contribution of seafood to total nutrients intake among Canadians. One limitation is the present analysis based on Day 1 recall only, which could not fully adjust the within-person variation in weekday/weekend food consumption. A single 24 h recall approach tends to overestimate intake in those who consumed fish on the day of the survey and return a zero intake for those fish eaters who did not consume fish on the survey day. Fish intake could be assessed more accurately with three to five consecutive 24 h dietary recalls or food frequency questionnaires. The average of Day 1 recall intakes is, however, an appropriate estimate of the average population’s usual intake [26]. Secondly, the total energy intake is lower in CCHS 2015. The comparison between 2004 and 2015 data may need to be interpreted with caution. Finally, the CCHS did not include data from the Indigenous Peoples living on reserve. Fish has always been an essential part of their traditional diets that contribute to their nutrition security [25,27].

## 7. Conclusions

Results of this study based on data collected from the CCHS can be used to guide the nutrition-related program and policy decisions among Canadians. Seafood is an excellent source of PUFAs, Vitamin A, Vitamin D, and Vitamin B12 for Canadians. Even though the fish consumption rate among Canadians was not as high as in other countries, the consumption of fish and seafood, particularly the environmentally sustainable species, can be promoted.

## Figures and Tables

**Table 1:** Sociodemographic characteristics of consumers and non-consumers of seafood among male and female Canadians in 2004 and 2015.

	2004						2015					
	Male (*n* = 13,753)		Female (*n* = 14,063)		Total (*n* = 27,816)		Male (*n* = 9167)		Female (*n* = 10,106)		Total (*n* = 19,273)	
	Consumer (%)	Non-Consumer (%)	Consumer (%)	Non-Consumer (%)	Consumer (%)	Non-Consumer (%)	Consumer (%)	Non-Consumer (%)	Consumer (%)	Non-Consumer (%)	Consumer (%)	Non-Consumer (%)
Age (years)	41.60 ± 0.62	35.60 ± 0.20	42.80 ± 0.71	37.62 ± 0.22	42.19 ± 0.48	36.57 ± 0.14	44.72 ± 0.84	40.29 ± 0.27	44.74 ± 0.81	42.19 ± 0.26	44.73 ± 0.55	41.25 ± 0.17
Total	15.80 ± 0.60	84.20 ± 0.60	17.01 ± 0.69	82.99 ± 0.69	16.38 ± 0.45	83.62 ± 0.45	17.43 ± 0.85	82.57 ± 0.85	17.99 ± 0.76	82.01 ± 0.76	17.71 ± 0.58	82.29 ± 0.58
Married												
No	13.50 ± 0.66	86.50 ± 0.66	14.93 ± 0.73	85.07 ± 0.73	14.22 ± 0.46	85.78 ± 0.46	16.74 ± 1.29	83.26 ± 1.29	16.32 ± 0.96	83.68 ± 0.96	16.52 ± 0.81	83.48 ± 0.81
Yes	18.01 ± 1.03	81.99 ± 1.03	19.30 ± 1.25	80.70 ± 1.25	18.61 ± 0.80	81.39 ± 0.80	18.02 ± 1.09	81.98 ± 1.09	19.67 ± 1.25	80.33 ± 1.25	18.83 ± 0.83	81.17 ± 0.83
Ethnicity												
Caucasian	14.34 ± 0.63	85.66 ± 0.63	14.91 ± 0.71	85.09 ± 0.71	14.62 ± 0.46	85.38 ± 0.46	15.36 ± 0.90	84.64 ± 0.9	15.51 ± 0.78	84.49 ± 0.78	15.43 ± 0.61	84.57 ± 0.61
Asian	26.61 ± 2.72	73.39 ± 2.72	38.18 ± 3.65	61.82 ± 3.65	31.97 ± 2.32	68.03 ± 2.32	23.66 ± 1.78	76.34 ± 1.78	24.41 ± 2.12	75.59 ± 2.12	24.05 ± 1.41	75.95 ± 1.41
Other	18.54 ± 2.77	81.46 ± 2.77	16.49 ± 2.43	83.51 ± 2.43	17.62 ± 1.83	82.38 ± 1.83	21.11 ± 3.41	78.89 ± 3.41	24.97 ± 3.69	75.03 ± 3.69	22.89 ± 2.51	77.11 ± 2.51
Education												
Secondary or below	12.89 ± 0.71	87.11 ± 0.71	14.24 ± 0.79	85.76 ± 0.79	13.55 ± 0.51	86.45 ± 0.51	15.26 ± 0.90	84.74 ± 0.90	16.69 ± 0.99	83.31 ± 0.99	15.95 ± 0.68	84.05 ± 0.68
Post-secondary	18.67 ± 1.03	81.33 ± 1.03	19.91 ± 1.19	80.09 ± 1.19	19.26 ± 0.75	80.74 ± 0.75	20.62 ± 1.59	79.38 ± 1.59	19.55 ± 1.29	80.45 ± 1.29	20.05 ± 1.03	79.95 ± 1.03
Coastal region												
No	16.21 ± 0.82	83.79 ± 0.82	14.76 ± 0.77	85.24 ± 0.77	15.51 ± 0.56	84.49 ± 0.56	15.82 ± 1.09	84.18 ± 1.09	17.36 ± 1.05	82.64 ± 1.05	16.61 ± 0.78	83.39 ± 0.78
Yes	15.30 ± 0.90	84.70 ± 0.90	19.66 ± 1.20	80.34 ± 1.20	17.42 ± 0.75	82.58 ± 0.75	19.50 ± 1.31	80.50 ± 1.31	18.82 ± 1.16	81.18 ± 1.16	19.16 ± 0.86	80.84 ± 0.86
Household income												
≤60,000	16.45 ± 0.81	83.55 ± 0.81	16.92 ± 0.85	83.08 ± 0.85	16.69 ± 0.60	83.31 ± 0.60	18.04 ± 1.92	81.96 ± 1.92	17.11 ± 1.41	82.89 ± 1.41	17.54 ± 0.87	82.46 ± 0.87
>60,000	15.01 ± 0.96	84.99 ± 0.96	17.14 ± 1.20	82.86 ± 1.20	15.98 ± 0.74	84.02 ± 0.74	17.25 ± 0.93	82.75 ± 0.93	18.29 ± 0.94	81.71 ± 0.94	17.83 ± 0.74	82.17 ± 0.74

Weighted mean ± SE for all values.

**Table 2:** Seafood consumption among Canadians across age and sex groups in 2004 and 2015.

	2004						2015					
	Male (*n* = 13,753)		Female (*n* = 14,063)		Total (*n* = 27,816)		Male (*n* = 9167)		Female (*n* = 10,106)		Total (*n* = 19,273)	
	Percentage of Consumer (%)	Average Consumption * (g)	Percentage of Consumer (%)	Average Consumption * (g)	Percentage of Consumer (%)	Average Consumption * (g)	Percentage of Consumer (%)	Average Consumption * (g)	Percentage of Consumer (%)	Average Consumption * (g)	Percentage of Consumer (%)	Average Consumption * (g)
2 to 3 years	12.74 ± 1.91	35.98 ± 5.53	13.85 ± 2.36	46.77 ± 7.68	13.29 ± 1.50	41.58 ± 4.79	21.74 ± 3.81	60.13 ± 8.04	17.66 ± 4.19	42.99 ± 7.37	19.73 ± 2.82	52.59 ± 6.00
4 to 8 years	10.89 ± 1.45	71.37 ± 8.71	10.43 ± 1.37	65.71 ± 9.30	10.67 ± 0.95	68.74 ± 6.19	12.65 ± 2.27	83.5 ± 16.91	12.58 ± 2.43	57.18 ± 10.57	12.61 ± 1.67	69.75 ± 10.04
9 to 13 years	10.56 ± 1.16	89.32 ± 11.83	10.02 ± 1.1	82.29 ± 9.47	10.30 ± 0.81	86.06 ± 7.77	10.30 ± 1.29	73.53 ± 6.56	11.65 ± 1.76	63.36 ± 9.28	10.96 ± 1.10	68.26 ± 5.74
14 to 18 years	10.12 ± 1.16	101.60 ± 11.38	9.17 ± 1.18	77.03 ± 7.66	9.68 ± 0.80	90.72 ± 7.28	9.54 ± 1.52	120.47 ± 13.8	15.74 ± 2.35	91.10 ± 10.56	12.64 ± 1.48	102.17 ± 7.71
19 to 30 years	12.46 ± 1.18	110.50 ± 14.24	17.59 ± 1.86	76.06 ± 6.53	14.73 ± 1.06	92.31 ± 7.31	12.58 ± 2.02	114.55 ± 14.8	17.48 ± 2.86	106.11 ± 21.92	14.89 ± 1.68	109.87 ± 12.87
31 to 50 years	16.61 ± 1.38	141.39 ± 12.76	19.01 ± 1.67	104.82 ± 9.59	17.76 ± 1.06	122.65 ± 8.12	18.79 ± 1.85	119.19 ± 7.83	18.50 ± 1.64	86.32 ± 6.69	18.64 ± 1.21	102.70 ± 5.17
51 to 70 years	22.84 ± 1.54	125.19 ± 8.82	19.09 ± 1.35	102.19 ± 7.36	20.96 ± 1.08	114.70 ± 5.68	22.22 ± 1.74	117.45 ± 6.58	20.12 ± 1.48	83.12 ± 4.76	21.14 ± 1.13	100.65 ± 4.26
≥71 years	18.32 ± 1.78	108.04 ± 9.44	21.75 ± 2.07	113.09 ± 12.44	20.27 ± 1.39	111.11 ± 7.99	17.09 ± 1.54	97.63 ± 6.05	19.00 ± 1.53	86.67 ± 5.66	18.16 ± 1.09	91.21 ± 4.30
Total	15.80 ± 0.60	120.17 ± 5.77	17.01 ± 0.69	95.95 ± 4.24	16.38 ± 0.45	108.02 ± 3.56	17.43 ± 0.85	111.40 ± 3.80	17.99 ± 0.76	84.86 ± 3.81	17.71 ± 0.58	97.69 ± 2.61

Weighted mean ± SE for all values. * Average consumption among consumers.

**Table 3:** Top 10 consumed seafood species among Canadians in 2004 and 2015.

	2004						2015					
	Male (*n* = 13,753)		Female (*n* = 14,063)		Total (*n* = 27,816)		Male (*n* = 9167)		Female (*n* = 10,106)		Total (*n* = 19,273)	
	Percentage of Consumer (%)	Average Consumption * (g)	Percentage of Consumer (%)	Average Consumption * (g)	Percentage of Consumer (%)	Average Consumption * (g)	Percentage of Consumer (%)	Average Consumption * (g)	Percentage of Consumer (%)	Average Consumption * (g)	Percentage of Consumer (%)	Average Consumption * (g)
Fish												
Salmon	3.54 ± 0.30	140.72 ± 13.52	4.01 ± 0.34	107.24 ± 8.18	3.76 ± 0.22	123.50 ± 7.70	5.37 ± 0.56	99.64 ± 6.63	5.04 ± 0.38	84.99 ± 5.88	5.20 ± 0.34	92.42 ± 4.35
Tuna	3.22 ± 0.29	82.19 ± 4.68	3.37 ± 0.32	66.08 ± 5.47	3.29 ± 0.21	74.22 ± 3.66	3.73 ± 0.49	70.29 ± 7.09	3.34 ± 0.33	53.45 ± 4.65	3.53 ± 0.30	62.19 ± 4.13
Cod	0.94 ± 0.19	134.97 ± 15.41	0.98 ± 0.15	83.11 ± 7.72	0.96 ± 0.12	109.40 ± 9.29	2.49 ± 0.33	95.18 ± 8.39	3.01 ± 0.42	58.65 ± 7.95	2.75 ± 0.26	74.85 ± 6.38
Trout	0.27 ± 0.06	122.03 ± 22.84	0.33 ± 0.07	105.35 ± 17.00	0.30 ± 0.05	113.13 ± 13.64	0.35 ± 0.10	84.26 ± 12.46	0.30 ± 0.08	95.50 ± 13.87	0.32 ± 0.06	89.60 ± 9.25
Sardine	0.33 ± 0.08	77.18 ± 8.46	0.14 ± 0.05	55.89 ± 23.32	0.24 ± 0.05	71.31 ± 8.60	0.28 ± 0.07	96.53 ± 21.46	0.10 ± 0.05	146.25 ± 64.27	0.19 ± 0.04	109.73 ± 25.67
Herring	0.15 ± 0.04	83.27 ± 11.38	0.14 ± 0.05	79.20 ± 16.09	0.15 ± 0.03	81.38 ± 9.01	0.11 ± 0.04	106.06 ± 24.02	0.08 ± 0.04	70.70 ± 21.42	0.09 ± 0.03	91.32 ± 17.25
Shellfish												
Shrimp	3.02 ± 0.28	56.78 ± 4.33	3.59 ± 0.42	45.35 ± 4.50	3.30 ± 0.24	50.76 ± 3.13	4.12 ± 0.53	37.65 ± 5.32	3.28 ± 0.34	34.84 ± 4.04	3.69 ± 0.31	36.38 ± 3.27
Crab	0.79 ± 0.16	122.38 ± 34.44	0.58 ± 0.12	64.40 ± 14.43	0.68 ± 0.10	98.81 ± 22.32	1.69 ± 0.39	35.00 ± 11.42	1.43 ± 0.26	44.25 ± 11.92	1.56 ± 0.24	39.31 ± 8.52
Scallop	0.54 ± 0.16	109.00 ± 39.38	0.50 ± 0.14	41.93 ± 8.47	0.52 ± 0.10	77.82 ± 24.46	0.54 ± 0.22	33.00 ± 13.85	0.65 ± 0.19	36.25 ± 12.42	0.60 ± 0.15	34.80 ± 8.61
Clam	0.31 ± 0.10	63.03 ± 13.99	0.23 ± 0.07	93.37 ± 26.29	0.27 ± 0.07	73.34 ± 14.17	0.33 ± 0.14	30.67 ± 17.46	0.15 ± 0.05	66.86 ± 13.80	0.24 ± 0.08	42.49 ± 13.55
Other species ^#^	4.88 ± 0.40	112.76 ± 6.84	5.57 ± 0.43	105.65 ± 7.89	4.59 ± 0.28	106.92 ± 5.74	6.22 ± 0.55	96.13 ± 7.92	5.64 ± 0.52	85.74 ± 6.48	5.92 ± 0.39	91.09 ± 5.08

* Average consumption among consumers. ^#^ Other species all combine.

**Table 4:** Daily energy and nutrients intake between consumers and non-consumers of seafood among Canadians in 2004 and 2015.

	2004						2015					
	Male (*n* = 13,753)		Female (*n* = 14,063)		Total (*n* = 27,816)		Male (*n* = 9167)		Female (*n* = 10,106)		Total (*n* = 19,273)	
	Consumer	Non-Consumer	Consumer	Non-Consumer	Consumer	Non-Consumer	Consumer	Non-Consumer	Consumer	Non-Consumer	Consumer	Non-Consumer
Energy (kcal)	2535.22 ± 43.59	2550.39 ± 21.64	1986.88 ± 33.56	1925.88 ± 13.28	2260.21 ± 28.24	2250.94 ± 13.39	2100.53 ± 46.42	2023.93 ± 16.87	1568.64 ± 28.83	1602.73 ± 13.59	1825.79 ± 28.51	1810.43 ± 11.70
Carbohydrates (g)	303.87 ± 5.16	319.07 ± 2.85	245.14 ± 4.98	247.61 ± 1.75	274.41 ± 3.67	284.8 ± 1.75	239.14 ± 4.98	247.29 ± 2.16	185.09 ± 3.84	201.69 ± 1.85	211.21 ± 3.18	224.17 ± 1.49
Fibre (g)	20.51 ± 0.48	19.12 ± 0.22	16.87 ± 0.39	16.13 ± 0.18	18.68 ± 0.32	17.68 ± 0.13	17.35 ± 0.42	17.60 ± 0.23	15.85 ± 0.50	15.66 ± 0.19	16.57 ± 0.32	16.61 ± 0.16
Total sugar (g)	119.85 ± 2.98	133.92 ± 1.56	102.11 ± 2.76	106.99 ± 1.06	110.95 ± 2.05	121 ± 0.96	93.67 ± 2.79	97.62 ± 1.18	70.72 ± 1.87	83.61 ± 1.00	81.81 ± 1.65	90.52 ± 0.78
Fat (g)	91.06 ± 2.17	93.31 ± 1.04	71.45 ± 1.74	70.54 ± 0.72	81.22 ± 1.40	82.38 ± 0.67	75.43 ± 2.12	74.39 ± 0.9	57.75 ± 1.34	58.86 ± 0.69	66.29 ± 1.26	66.51 ± 0.61
Saturated fat (g)	27.41 ± 0.71	31.77 ± 0.38	22.29 ± 0.56	24.28 ± 0.31	24.84 ± 0.46	28.17 ± 0.25	23.82 ± 0.81	24.61 ± 0.34	17.83 ± 0.47	19.99 ± 0.27	20.72 ± 0.47	22.26 ± 0.23
PUFA (g)	18.00 ± 0.53	15.48 ± 0.22	13.77 ± 0.43	11.9 ± 0.14	15.87 ± 0.34	13.76 ± 0.13	16.55 ± 0.47	15.19 ± 0.23	13.16 ± 0.35	12.19 ± 0.21	14.79 ± 0.29	13.66 ± 0.16
Cholesterol (mg)	366.98 ± 15.88	318.28 ± 5.53	300.71 ± 12.77	235.74 ± 3.64	333.74 ± 11.35	278.7 ± 3.34	328.26 ± 10.46	281.62 ± 5.32	247.34 ± 10.03	210.92 ± 3.82	286.46 ± 7.63	245.78 ± 3.41
Protein (g)	112.70 ± 2.54	99.64 ± 1.07	90.68 ± 2.39	75.06 ± 0.69	101.65 ± 1.79	87.85 ± 0.65	95.93 ± 2.61	83.33 ± 0.84	70.32 ± 1.50	65.00 ± 0.63	82.69 ± 1.50	74.03 ± 0.53
Vitamin A (µg)	919.07 ± 117.34	783.69 ± 15.51	718.65 ± 20.49	690.97 ± 11.72	818.55 ± 59.36	739.22 ± 10	738.34 ± 29.14	640.6 ± 14.52	625.31 ± 23.97	599.52 ± 15.08	679.95 ± 19.11	619.77 ± 10.54
Vitamin D (µg)	12.08 ± 0.79	6.01 ± 0.08	10.53 ± 0.82	5.02 ± 0.07	11.30 ± 0.56	5.53 ± 0.05	9.63 ± 0.49	4.38 ± 0.08	7.35 ± 0.34	3.65 ± 0.07	8.45 ± 0.30	4.00 ± 0.05
Vitamin C (µg)	158.28 ± 6.11	145.99 ± 2.56	145.87 ± 4.42	130.99 ± 1.93	152.05 ± 3.80	138.79 ± 1.57	105.6 ± 4.21	102.90 ± 2.60	99.60 ± 5.01	96.76 ± 2.02	102.50 ± 3.36	99.78 ± 1.63
Niacin (µg)	52.53 ± 1.14	45.56 ± 0.49	42.69 ± 1.36	34.03 ± 0.32	47.59 ± 0.90	40.03 ± 0.3	47.77 ± 1.39	41.09 ± 0.45	34.66 ± 0.84	31.58 ± 0.34	40.99 ± 0.79	36.26 ± 0.28
Vitamin B6 (µg)	2.39 ± 0.06	2.15 ± 0.03	1.99 ± 0.06	1.65 ± 0.02	2.19 ± 0.04	1.9 ± 0.01	2.02 ± 0.06	1.75 ± 0.03	1.53 ± 0.04	1.39 ± 0.02	1.76 ± 0.03	1.56 ± 0.01
Vitamin B12 (µg)	8.49 ± 0.47	4.69 ± 0.11	7.03 ± 0.51	3.44 ± 0.08	7.76 ± 0.33	4.09 ± 0.06	6.56 ± 0.26	3.99 ± 0.09	5.18 ± 0.3	3.09 ± 0.08	5.84 ± 0.20	3.53 ± 0.06
Natural occurring folate (µg)	295.62 ± 8.09	251.53 ± 2.71	247.79 ± 5.36	218.50 ± 3.44	271.63 ± 4.73	235.69 ± 2.14	231.94 ± 5.69	211.38 ± 2.82	204.27 ± 6.49	185.64 ± 2.81	217.64 ± 4.28	198.33 ± 2.05
Phosphorus (mg)	1811.98 ± 37.51	1589.72 ± 13.36	1512.09 ± 33.06	1275.62 ± 9.24	1661.58 ± 25.18	1439.11 ± 8.37	1600.25 ± 39.06	1324.41 ± 12.78	1228.17 ± 29.56	1089.25 ± 10.83	1408.05 ± 24.40	1205.21 ± 9.00
Magnesium (mg)	420.96 ± 8.81	362.76 ± 3.14	347.83 ± 6.02	302.25 ± 2.26	384.28 ± 5.34	333.74 ± 1.9	353.58 ± 7.56	311.25 ± 3.30	290.16 ± 7.01	262.23 ± 2.48	320.81 ± 5.21	286.4 ± 2.20
Iron (mg)	17.50 ± 0.32	16.91 ± 0.16	13.89 ± 0.32	13.15 ± 0.1	15.69 ± 0.23	15.1 ± 0.09	12.92 ± 0.28	13.45 ± 0.15	10.50 ± 0.25	10.79 ± 0.11	11.67 ± 0.19	12.10 ± 0.09
Zinc (mg)	13.25 ± 0.37	13.62 ± 0.15	10.66 ± 0.29	10.23 ± 0.09	11.94 ± 0.25	11.99 ± 0.09	11.75 ± 0.53	11.32 ± 0.15	8.15 ± 0.23	8.74 ± 0.09	9.88 ± 0.29	10.01 ± 0.09
Sodium (g)	3.74 ± 0.09	3.77 ± 0.04	3.10 ± 0.08	2.84 ± 0.03	3.42 ± 0.06	3.32 ± 0.03	3.17 ± 0.09	2.95 ± 0.04	2.40 ± 0.06	2.27 ± 0.02	2.78 ± 0.05	2.60 ± 0.02
Potassium (g)	3.82 ± 0.07	3.52 ± 0.03	3.28 ± 0.06	2.89 ± 0.02	3.55 ± 0.05	3.22 ± 0.02	3.11 ± 0.07	2.75 ± 0.03	2.47 ± 0.05	2.35 ± 0.02	2.78 ± 0.04	2.55 ± 0.02
DHA (g)	--	--	--	--	--	--	0.51 ± 0.04	0.04 ± 0.01	0.46 ± 0.05	0.03 ± 0.01	0.48 ± 0.03	0.03 ± 0.01
EPA (g)	--	--	--	--	--	--	0.29 ± 0.03	0.01 ± 0.01	0.26 ± 0.04	0.01 ± 0.01	0.27 ± 0.02	0.01 ± 0.01

--: not available. PUFA: polyunsaturated fatty acids; DHA: docosahexaenoic acid; EPA: eicosapentaenoic acid.

**Table 5:** Contribution of daily energy and nutrients intake from seafood (%).

	2004						2015					
	Male ≤18	Male 19+	Female ≤18	Female 19+	Total ≤18	Total 19+	Male ≤18	Male 19+	Female ≤18	Female 19+	Total ≤18	Total 19+
Total weight	0.30	0.66	0.29	0.62	0.29	0.63	0.41	0.71	0.41	0.67	0.41	0.69
Energy (kcal)	0.46	1.33	0.47	1.45	0.46	1.38	0.61	1.36	0.67	1.46	0.63	1.40
Carbohydrates	0.02	0.04	0.01	0.05	0.01	0.04	0.02	0.05	0.02	0.06	0.02	0.05
Fibre	0.01	0.01	0.01	<0.01	<0.01	<0.01	<0.01	0.01	0.01	0.01	<0.01	0.01
Total sugar	0.01	<0.01	<0.01	<0.01	<0.01	<0.01	0.01	0.02	0.01	0.02	0.01	0.02
Fat	0.35	1.04	0.36	1.13	0.35	1.08	0.54	1.16	0.64	1.26	0.59	1.20
Cholesterol	2.20	4.95	2.20	5.62	2.19	5.24	3.32	5.65	3.06	6.30	3.19	5.95
Protein	2.15	5.48	2.27	6.01	2.19	5.71	2.69	5.42	2.90	5.91	2.78	5.65
Vitamin D	4.55	17.47	4.56	18.63	4.55	18.03	6.40	17.22	8.15	18.49	7.2	17.84
Niacin	2.33	5.49	2.53	6.29	2.41	5.84	2.86	5.39	3.27	5.95	3.04	5.65
Vitamin B6	1.20	3.29	1.36	3.59	1.26	3.42	2.24	4.14	2.54	4.35	2.38	4.24
Vitamin B12	8.35	16.96	6.87	19.07	7.73	17.91	6.00	14.99	7.77	16.94	6.8	15.91
Phosphorus	1.48	4.84	1.53	5.03	1.50	4.92	2.35	5.24	2.53	5.57	2.43	5.40
Magnesium	1.04	2.56	1.05	2.49	1.04	2.52	1.21	2.32	1.25	2.33	1.22	2.32
Iron	0.89	1.96	0.73	2.14	0.81	2.04	0.58	1.41	0.70	1.51	0.63	1.45
Zinc	1.14	1.99	1.88	2.94	1.45	2.42	0.96	2.78	1.09	2.23	1.02	2.52
Sodium	0.50	1.68	0.54	1.61	0.51	1.65	0.96	1.98	0.96	2.26	0.95	2.11
Potassium	0.89	2.44	0.89	2.35	0.89	2.39	1.22	2.55	1.26	2.53	1.23	2.54
DHA	--	--	--	--	--	--	62.45	72.86	65.22	76.61	63.78	74.73
EPA	--	--	--	--	--	--	25.36	46.75	25.31	27.72	25.91	26.52

Total energy and nutrients intake from food sources, supplement not included. --: not available. DHA: docosahexaenoic acid; EPA: eicosapentaenoic acid.

## Data Availability

Data was accessed at the Carleton, Ottawa, Outaouais Research Data Center (COOL RDC), which is part of the Canadian Research Data Centre Network (CRDCN).

## References

[1] Benjamin E.J., Virani S.S., Callaway C.W., Chamberlain A.M., Chang A.R., Cheng S., Chiuve S.E., Cushman M., Delling F.N., Deo R. (2018). Heart disease and stroke statistics—2018 update: A report from the American Heart Association. Circulation.

[2] Kris-Etherton P.M., Harris W.S., Appel L.J. (2002). Fish consumption, fish iil, Omega-3 fatty acids, and cardiovascular disease. Circulation.

[3] Rimm E.B., Appel L.J., Chiuve S.E., Djoussé L., Engler M.B., Kris-Etherton P.M., Mozaffarian D., Siscovick D.S., Lichtenstein A.H. (2018). Seafood long-chain *n-*3 Polyunsaturated Fatty Acids and cardiovascular disease: A science advisory from the American Heart Association. Circulation.

[4] Mozaffarian D., Wu J.H. (2011). Omega-3 fatty acids and cardiovascular disease. J. Am. Coll. Cardiol..

[5] Dunstan J.A., Simmer K., Dixon G., Prescott S.L. (2008). Cognitive assessment of children at age 21/2 years after maternal fish oil supplementation in pregnancy: A randomised controlled trial. Arch. Dis. Child. Fetal Neonatal Ed..

[6] Daniels J.L., Longnecker M.P., Rowland A.S., Golding J. (2004). Fish intake during pregnancy and early cognitive development of offspring. Epidemiology.

[7] Department of Health and Human Services, U.S. Department of Agriculture 2015–2020 Dietary Guidelines for Americans. 8th Edition. December 2015. https://health.gov/our-work/food-and-nutrition/2015-2020-dietary-guidelines/.

[8] Health Canada Eating Well with Canada’s Food Guide 2019. https://food-guide.canada.ca/en/.

[9] Health Canada Eating Well with Canada’s Food Guide 2007. https://food-guide.canada.ca/en/.

[10] Statistics Canada Table 11-10-0125-01 Detailed Food Spending, Canada, Regions and Provinces.

[11] Fisheries and Oceans Canada (2018). Outlook to 2027 for Canadian Fish and Seafood. https://waves-vagues.dfo-mpo.gc.ca/Library/40732836.pdf.

[12] Health Canada Human Health Risk Assessment of Mercury in Fish and Health Benefits of Fish Consumption. Ottawa: 2007. https://www.canada.ca/en/health-canada/services/food-nutrition/reports-publications/human-health-risk-assessment-mercury-fish-health-benefits-fish-consumption.html.

[13] Béland Y. (2002). Canadian community health survey—Methodological overview. Health Rep..

[14] Statistics Canada (2008). Canadian Community Health Survey, Cycle 2.2 (2004), Nutrition—User Guide for General Health (including Vitamin & Mineral Supplements) & 24-Hour Dietary Recall Components.

[15] Health Canada (2015). Canadian Nutrient File (CNF).

[16] Kirkpatrick S.I., Raffoul A., Lee K.M., Jones A.C. (2019). Top dietary sources of energy, sodium, sugars, and saturated fats among Canadians: Insights from the 2015 Canadian Community Health Survey. Appl. Physiol. Nutr. Metab..

[17] Love D.C., Asche F., Conrad Z., Young R., Harding J., Nussbaumer E.M., Thorne-Lyman A.L., Neff R. (2020). Food sources and expenditures for seafood in the United States. Nutrients.

[18] Farmery A.K., Hendrie G.A., O’Kane G., McManus A., Green B.S. (2018). Sociodemographic Variation in Consumption Patterns of Sustainable and Nutritious Seafood in Australia. Front. Nutr..

[19] Moya J. (2004). Overview of fish consumption rates in the United States. Hum. Ecol. Risk Assess. Int. J..

[20] Terry A.L.A., Herrick K., Afful J., Ahluwalia N. (2018). Seafood Consumption in the United States, 2013–2016.

[21] Murakami K., Livingstone M.B.E., Sasaki S. (2018). Thirteen-year trends in dietary patterns among Japanese adults in the National Health and Nutrition Survey 2003–2015: Continuous westernization of the Japanese diet. Nutrients.

[22] Fabinyi M. (2016). Sustainable seafood consumption in China. Mar. Policy.

[23] Statistics Canada (2003). Food Statistics 2002.

[24] Kenny T.-A., Hu X.F., Kuhnlein H., Wesche S.D., Chan H.M. (2018). Dietary sources of energy and nutrients in the contemporary diet of Inuit adults: Results from the 2007–08 Inuit Health Survey. Public Health Nutr..

[25] Chan L., Bata M., Sadik T., Tikhonov C., Schwartz H., Fediuk K., Ing A., Marushka L., Lindhorst K., Barwin L. (2019). FNFNES Final Report for Eight Assembly of First Nations Regions: Draft Comprehensive Technical Report.

[26] Tugault-Lafleur C.N., Black J.L. (2019). Differences in the quantity and types of foods and beverages consumed by Canadians between 2004 and 2015. Nutrients.

[27] Marushka L., Kenny T.-A., Batal M., Cheung W.W.L., Fediuk K., Golden C.D., Salomon A.K., Sadik T., Weatherdon L.V., Chan H.M. (2019). Potential impacts of climate-related decline of seafood harvest on nutritional status of coastal First Nations in British Columbia, Canada. PLoS ONE.

